# Recombinant protein subunit vaccine booster following two-dose inactivated vaccines dramatically enhanced anti-RBD responses and neutralizing titers against SARS-CoV-2 and Variants of Concern

**DOI:** 10.1038/s41422-021-00590-x

**Published:** 2021-11-23

**Authors:** Jingwen Ai, Haocheng Zhang, Qiran Zhang, Yi Zhang, Ke Lin, Zhangfan Fu, Jieyu Song, Yuanhan Zhao, Mingxiang Fan, Hongyu Wang, Chao Qiu, Yang Zhou, Wenhong Zhang

**Affiliations:** 1 Department of Infectious Diseases, National Medical Center for Infectious Diseases, Shanghai Key Laboratory of Infectious Diseases and Biosafety Emergency Response, Huashan Hospital, Shanghai Medical College, Fudan University, Shanghai, China; 2 National Clinical Research Center for Aging and Medicine, Huashan Hospital, Fudan University, Shanghai, China; 3 Key Laboratory of Medical Molecular Virology (MOE/MOH) Shanghai Medical College, Fudan University, Shanghai, China

**Keywords:** Immunology, Biological techniques

Dear Editor,

As of October, 2021, SARS-CoV-2 has infected more than 230 million people; promoting roll-out vaccinations could help build herd immunity for the pandemic. However, the waning of antibody in magnitude and decreased protective efficacy in multiple types of vaccines have been reported. Along with the circulating variants to some degree escaping from immune response, these led to the argument of further booster vaccination in recipients who have previously received “priming” vaccination. Recent studies reported that a third homologous dose showed a satisfying safety profile and higher immune response, including BNT162b2 mRNA vaccine, CoronaVac inactivated vaccine and BBIBP-CorV vaccine.^1–3^ Additionally, heterologous prime-boost vaccination of ChAdOx1 nCoV-19 followed by BNT162b2 induced a higher neutralizing activity compared to two homologous doses of ChAdOx1 nCoV-19.^4^ Until now, the effect of heterologous vaccination of recombinant protein subunit vaccine primed with two doses of inactivated vaccines has not been evaluated, and data along this line could provide further evidence in establishing future global boosting strategies.

We conducted this prospective, open-label study in a single center (Huashan Hospital, National Medical Center for Infectious Diseases (NMCID), Shanghai, China) to explore the safety and immunogenicity of a third boosting vaccination shot using 25 μg of protein subunit vaccine (ZF2001) administered at an interval of 4–8 months following previous priming vaccination by two doses of inactivated whole-virion vaccines (CoronaVac or BBIBP-CorV) in healthy adults. Safety was assessed by observing and analyzing adverse reactions, and plasma surrogate virus neutralization test (sVNT) was used for determining neutralizing activity. Pseudoviruses incorporating the spike (S) protein from Wuhan-Hu-1 and four Variants of Concern (VOCs) were constructed using DNA plasmid expressing S protein and G*∆G-VSV (VSV G pseudovirus) in 293T cells, and plasma pseudovirus neutralization test (pVNT) was conducted to detect neutralizing titers.^5^ Anti-RBD response was assessed using superparamagnetic microparticle based direct chemiluminescence immunoassay. SARS-CoV-2 S and nucleocapsid (N) protein-specific cellular immune responses were analyzed using IFN-γ release assay to assess the immunogenicity. The details of the study protocol are described in the Supplementary Information.

In the baseline characteristics study, 71 participants of booster group and 51 of control group were included (Supplementary information, Fig. S1). The median age was 28.00 (interquartile range (IQR) 25.00–44.30) in booster group and 26.00 (24.00–52.00) in control group (*P* = 0.9880). Thirty-one (43.70%) participants in booster group and 22 (43.10%) in control group were male (*P* = 0.9540). Body mass index was slightly lower in booster group than control group (22.30 vs 23.40, *P* = 0.0390). The demographic characteristics were detailed in Supplementary information, Table S1.

As expected, the baseline immune responses 4–8 months after the “priming” two-dose inactivated vaccination were comparable between the two groups. The median IFN-γ spot forming units (SFU) per million peripheral blood mononuclear cell (PBMC) against S1, S2, N peptide were 5 (IQR 0–10), 0 (0–10) and 15 (5–35) in control group, and 10 (0–20), 5 (0–20) and 10 (0–25) in booster group, respectively (Fig. 1a). Next, we evaluated the neutralizing antibody level and anti-RBD antibody level. Geometric mean titers (GMTs) of sVNT and pVNT were 18.19 (95% CI, 13.12–25.20) and 24.89 (20.63–30.02), and the median of total anti-RBD antibody and IgG level was 8.47 binding antibody units (BAU)/mL (IQR 2.84–30.06) and 4.42 (2.09–12.41) in the booster group, respectively (Fig. 1b–e).

**Fig. 1:**
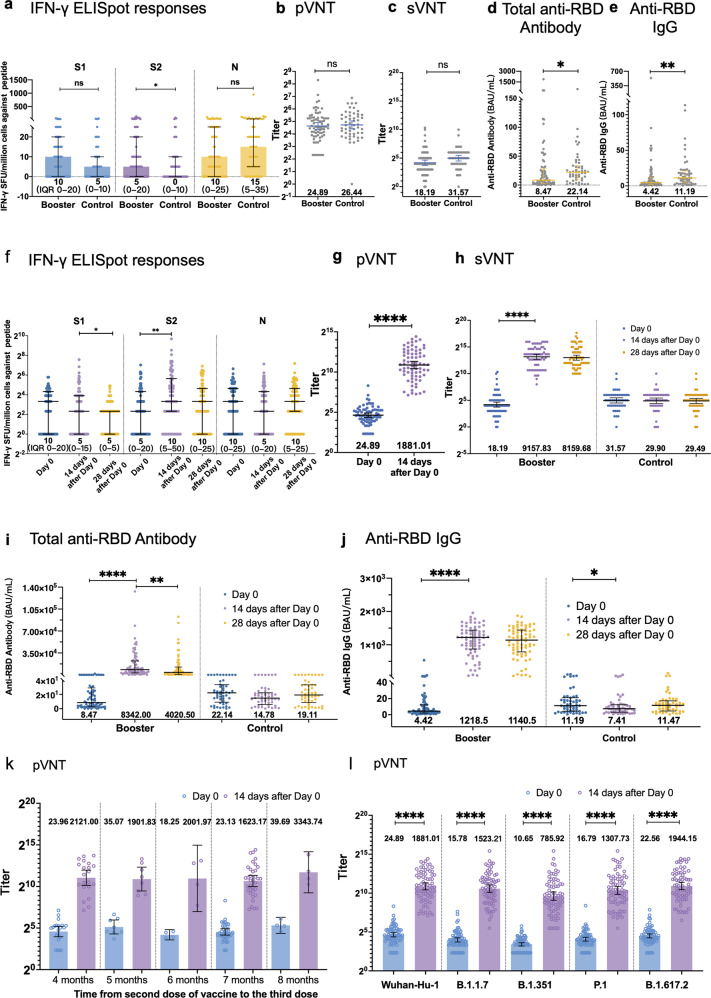
Immune response against SARS-CoV-2 and Variants of Concern after recombinant protein subunit vaccine booster following two-dose inactivated vaccines. **a** Baseline IFN-γ SFU/million PBMCs against S1, S2 and N peptide at 4–8 months after the second dose. **b** Baseline antibody response 4–8 months after second dose evaluated by pVNT. **c** Baseline antibody response 4–8 months after second dose evaluated by sVNT. **d** Baseline antibody response 4–8 months after second dose evaluated by total anti-RBD antibody. **e** Baseline antibody response 4–8 months after second dose evaluated by anti-RBD IgG. **f** IFN-γ SFU/million PBMCs against S1, S2 and N peptide in booster group. The IFN-γ -producing T cell spots were counted on the day of third dose, 14 days after third dose and 28 days after third dose. **g** Antibody response in boost group evaluated by pVNT. **h** Antibody response in boost group evaluated by sVNT. **i** Antibody response in boost group evaluated by total anti-RBD antibody. **j** Antibody response in boost group evaluated by anti-RBD IgG. **k** Pseudovirus neutralizing responses by various intervals between previous two vaccinations and the third booster vaccination. **l** Pseudovirus neutralizing responses after previous two vaccinations and the third booster vaccination against Wuhan-Hu-1 and VOCs, including, B.1.1.7 (Alpha Variant), B.1.351 (Beta Variant), P.1 (Gamma Variant) and B.1.617.2 (Delta Variant) strains. Samples with undetectable levels of IFN-γ SFU/million PBMCs were assigned a value of 2^0^ IFN-γ SFU/million PBMCs. Samples with undetectable levels of sVNT were assigned a value of 2^0^ titer.

In the analysis of immune response post booster vaccination, T cell response increase is overall weak. A significant increase in IFN-γ SFU per million PBMCs was observed on day 14 against S2 peptide (10 vs 5, *P* = 0.0029) while no obvious increase was found in S1 and N peptide pools (Fig. 1f).

On day 14 post booster vaccination, levels of pVNT were 75.58 times (geometric mean fold ratio (GMFR), 95% CI 61.47–92.91) of the baseline levels (Fig. 1g). By day 14, sVNT geometric mean titers (GMTs) increased to 503.47 times of the baseline levels (339.63–746.28), and by day 28 to 448.60 times of the GMTs at baseline (292.21–688.81) (Fig. 1h). Median anti-RBD antibody level and IgG level were 8342.00 BAU/mL (IQR 3223.87-22350.00) and 1218.50 BAU/mL (869.75–1444.00) on day 14, respectively, which are dramatically higher than the baseline antibody levels (Fig. 1i, j). By day 28, the total anti-RBD antibody level (4020.50 BAU/mL, 950.30–13050.00) and IgG level (1140.50 BAU/mL, 786.60–1446.00) decreased a bit from the peak level (Fig. 1i, j). The control group exhibited a stable profile of low antibody titers.

Antibody levels at 14 days after the third dose showed no significant difference among regimens with various intervals between the second and third shots (pVNT GMFR of 88.51 (95% CI, 68.83–113.82) for 4-month; 54.23 (34.54–85.17) for 5-month, 109.71 (9.78–1231.12) for 6-month; 70.19 (47.44–103.85) for 7-month; 84.25 (28.73–247.06) for 8-month interval) (Fig. 1k). Additionally, these booster doses induced at least 70-fold increase in neutralizing GMT levels against four variant pseudoviruses compared to the baseline level (pVNT GMFR of 96.54 (74.64–124.82) for Alpha; 73.79 (52.38–103.94) for Beta, 77.91 (55.72–108.94) for Gamma; and 86.16 for Delta (64.89–114.39), *P* < 0.0001) (Fig. 1l). Univariate analysis of factors associated with high neutralizing titers (top 30%) at day 14 of boosters revealed no factor with statistical significance (Supplementary information, Table S2), thus multivariate analysis was not further performed.

In the booster group, solicited injection site and systemic adverse reactions were reported by 30 (42.30%) and 8 (11.30%) participants within 3 days after the boosting dose. The most common injection site and systemic adverse reaction was pain (20, 28.20%) and fatigue (6, 8.50%). All adverse events were mild or moderate and mostly disappeared 3 days after the booster vaccination. Only 4 (5.60%) and 1 (1.40%) participant reported emerging or persisting injection site adverse reactions from day 4 to 14 and from day 15 to 28, respectively, and 2 (2.80%) and 1 (1.40%) reported solicited systemic adverse reactions in these periods. Unsolicited systemic adverse events were reported by 5 (7.00%) participants, and none of unsolicited local adverse events were reported (Supplementary information, Table S3).

Our study found that 4–8 months post the two-dose vaccination, participant’s neutralizing GMTs were still detectable, but decreased compared to 14 days post the second vaccination shot (data from Huashan Hospital; Ethics Number: KY2021-041), and the IFN-γ production by specific T cells was also detectable but lower than 12 weeks after the second vaccination shot.^6^ A third booster dose following previous two-dose inactivated vaccines could significantly recall and increase functional B cell responses by 27- to 75-fold compared to those 14 days post the second vaccination shot for all tested VOCs (Supplementary information, Fig. S2). These findings suggested that the “priming” of two-dose inactivated vaccines in our study could elicit durable humoral and cellular immunity, which could be successfully recalled by a third booster dose to provide protection against SARS-CoV-2 and its variants. However, the neutralizing antibody decay rate and long-term B and T cell memory levels still need to be evaluated in the follow-up study.

Immunogenicity of heterologous vaccination of protein subunit vaccine primed with inactivated vaccines has not been evaluated before the current study, though a few third dose homologous and heterologous booster studies have been conducted. The phase 1 and 2 clinical trials of three doses of protein subunit vaccine showed that neutralizing GMTs had a 5.6-fold increase after the third dose compared to the second in the 25 μg group.^7^ A third homologous dose of 30 μg BNT162b2 injection around 8 months after the second dose showed a 5- to 7-fold increase in the GMTs.^1^ Homologous BBIBP booster study showed 1- to 3-fold increase after the third dose (day 56) compared to the GMT level of around 80 after the second dose (day 28).^3^ Homologous CoronaVac booster study uncovered an ~3-fold increase of GMT level from the second dose to the third dose.^2^ After 14 days of homologous ChAdOx1 nCoV-19 boost, the GMT levels increased to 3.69 times as high as the levels at baseline.^8^ For heterologous booster study, a third Convidecia shot primed with two-dose CoronaVac induced a 78-fold increase in neutralizing antibody levels (197.4 vs 2.5).^9^ These studies, combined with our results, demonstrated that heterologous prime-boost may lead to higher fold increase of neutralizing GMTs than homologous boost. Our study showed that heterologous vaccination of protein subunit vaccine could also enhance the response against SARS-CoV-2 variants, which may be explained by that the third dose of vaccination could recall T and B memory cells, leading to a rebound in immune responses.

To address the relationship between levels of immune response and the real-world protection efficacy of the vaccines is crucial for the future establishment of the booster strategy. In our study, the median level of anti-RBD IgG was about 1140.00 BAU/mL 28 days after the third dose, which was higher than 506.00 BAU/mL (the threshold published previously that correlated with a reduced risk of symptomatic infection).^10^ Since the probability of infection decreases on average with higher immune responses, the third-dose booster vaccination strategy may be an efficient method for enhancing the protection of high-risk groups.

Previous study found that antibody levels after the second vaccination shot were higher among those with longer intervals between doses. However, we found no significant difference in the levels of antibody responses among 4–8 months of intervals to the third dose in our study. Due to the limited sample size, more data are required to generalize for the larger population to optimize the time-frame for the third booster dose.

Our study revealed satisfactory safety data, and previous trials reported 48% mild or moderate adverse events during the whole schedule of three-dose protein subunit vaccination in healthy adults,^7^ similar to our results. We suggest that after two doses of inactivated vaccines as priming, a third dose of recombinant protein subunit vaccine as booster, might be safe for healthy adults aged 18–59 years. As the participants in our study received different priming vaccination (either two doses of CoronaVac or BBIBP-CorV), we further performed subgroup analysis of participants receiving different priming vaccinations, and all the subgroup analysis were in consistency with the whole group analysis, as shown in the Supplementary Dataset. Nevertheless, further researches are needed before our results can be applied to the elderly, children, adolescents, and populations with coexisting chronic diseases or immunocompromised history, who were reported to be more vulnerable to SARS-CoV-2 infection. Efficacy of the vaccines, regarding breakthrough SARS-CoV-2 infections, COVID-19 morbidity and mortality following the boosting dose vaccination, which was not assessed in this study due to the limited cases in China, should be further evaluated in populations with higher risk of exposure. The participants in this study will be followed up to 12 months after the booster vaccination, and persistency of the enhanced immunity against SARS-CoV-2 and VOCs induced by the booster vaccination will be evaluated in the future.

In conclusion, after two doses of inactivated whole-virion vaccines as the “priming” shot, a third heterologous booster of protein subunit vaccine was safe and highly immunogenic for healthy adults, which significantly recalled and increased immune responses against SARS-CoV-2 and its variants. Our findings provide an important piece of evidence for establishing future global heterologous boosting strategy against COVID-19.

## Supplementary information

Supplementary Information
