# Supplementary material for: Recombinant protein subunit vaccine booster following two-dose inactivated vaccines dramatically enhanced anti-RBD responses and neutralizing titers against SARS-CoV-2 and Variants of Concern
Source: Cell Res. 2021 Nov 23;32(1):103–6. doi: 10.1038/s41422-021-00590-x (PMC8609258; doi:10.1038/s41422-021-00590-x)
Supplement: Supplementary file 1 — Supplementary Information [file 41422_2021_590_MOESM1_ESM.pdf]

Supplementary materials

Supplementary information, Fig. S1. Flowchart of study

Supplementary information, Fig. S2. The neutralizing titer by 14 days after second vaccination shots in part of participants

Supplementary information, Table S1. Basic Characteristics of Participants

Supplementary information, Table S2. Factors for higher neutralizing responses to the third-booster vaccination shots

Supplementary information, Table S3. Solicited and unsolicited adverse reactions

Supplementary methods. Study protocol

Supplementary Dataset. Subgroup analysis of participants received priming vaccination by two doses of CoronaVac or BBIBP-CorV

Supplementary information, Fig. S1. Flowchart of study

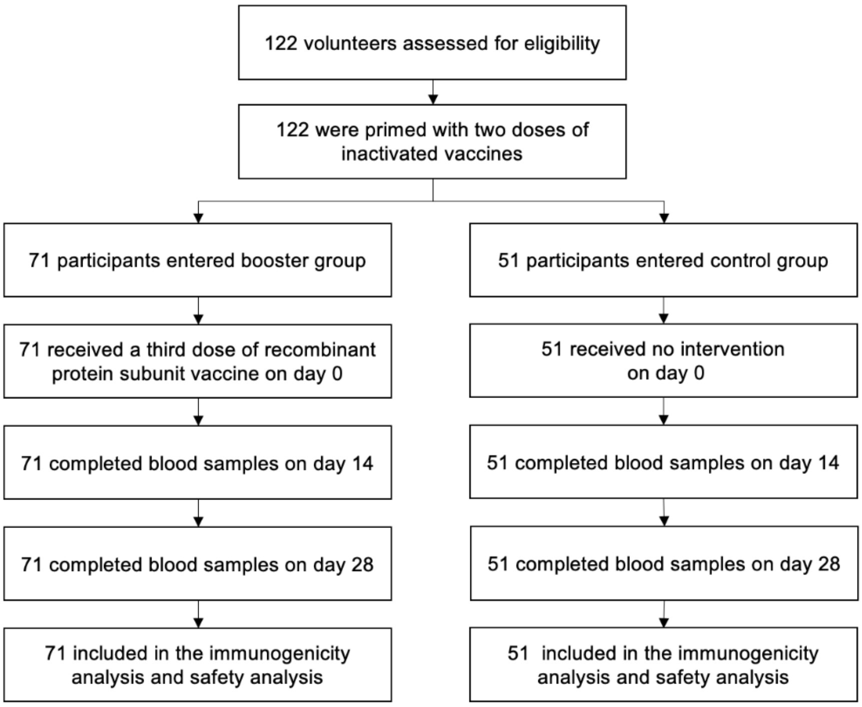

Supplementary information, Fig. S2. The neutralizing titer by 14 days after second vaccination shots in part of participants

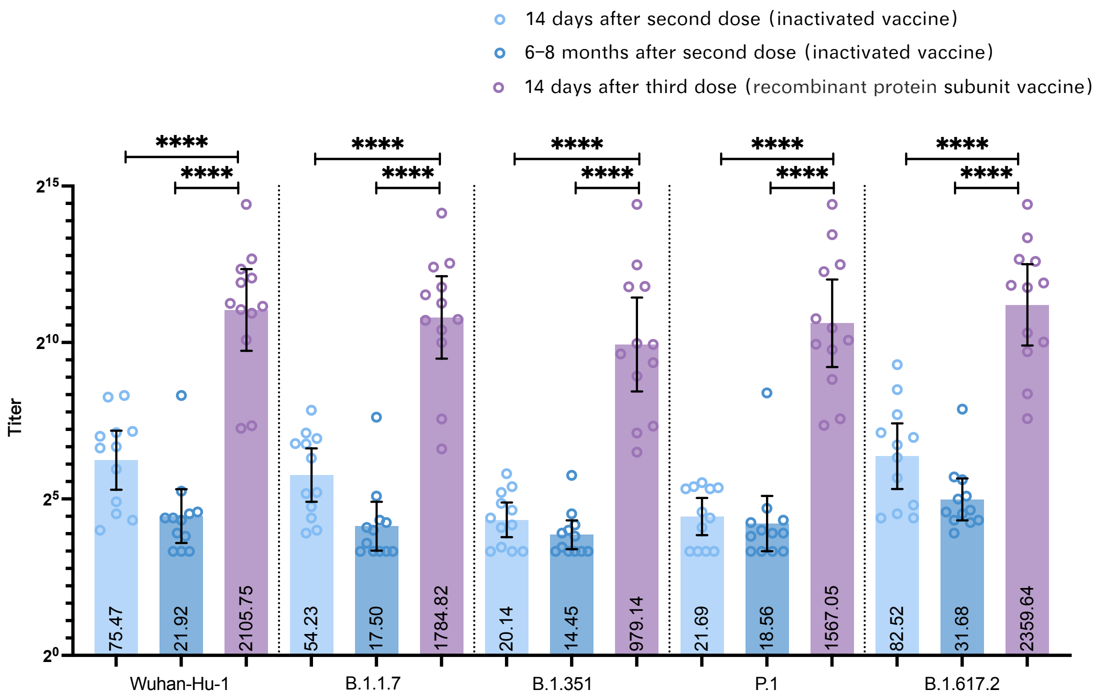

Supplementary information, Table S1. Basic Characteristics of Participants

	Booster Group (n=71)	Control Group (n=51)	P value
**Age (years)**	28.0 (25.0-44.3)	26.0 (24.0-52.0)	0.988
**Sex, male (%)**	31 (43.7%)	22 (43.1%)	0.954
**BMI**	22.3 (20.3-25.2)	23.4 (20.8-25.9)	**0.039**
**Ethnicity, Asian**	71 (100%)	51 (100%)	**-**
**Comorbidities**			
Any (%)	9 (12.7%)	6 (11.8%)	0.558
CAD, (%)	0 (0.0%)	0 (0.0%)	0.999
HTN, (%)	2 (2.8%)	5 (9.8%)	0.128
DM, (%)	0 (0.0%)	1 (2.0%)	0.999
Arrhy, (%)	1 (1.4%)	0 (0.0%)	0.999
NASH, (%)	1 (1.4%)	0 (0.0%)	0.999
Asthma, (%)	1 (1.4%)	0 (0.0%)	0.999
Rhinitis, (%)	2 (2.8%)	0 (0.0%)	0.999
Goiter, (%)	1 (1.4%)	0 (0.0%)	0.999
Urticaria, (%)	1 (1.4%)	0 (0.0%)	0.999

Note

Date presented as: Median (interquartile) or number (percentage).

For all categorical variables, the Chi-Square statistic was used.

Continuous variables were compared by using a Mann-Whitney test.

$P < 0.05$ was considered statistically significant for all analyses.

BMI= Body Mass Index, CAD=Coronary artery disease, HTN=Hypertension, DM= Diabetes mellitus, Arrhy=Arrhythmia, NASH=non-alcoholic steatohepatitis.

Supplementary information, Table S2. Factors for higher neutralizing responses to the third-booster vaccination shots

	**High neutralizing titer group (n=24)**	**Low neutralizing titer group (n=22)**	**OR**	**P value**
**Age (years)**	29.0 (24.5-40.8)	35.0 (26.8-45.3)	-	0.218
**Sex, male (%)**	10 (41.7%)	15 (68.2%)	0.33 (0.099-1.12)	0.071
**BMI**	21.1 (20.2-25.0)	23.5 (20.9-25.6)	-	0.368
**Overweight, (%)**	6 (25.0%)	8 (36.4%)	0.529 (0.146-1.925)	0.334
**Any comorbidity**	2 (8.3%)	1 (4.5%)	1.909 (0.161-22.656)	0.608

Note

Date presented as: Median (interquartile) or number (percentage).

For all categorical variables, the Chi-Square statistical was used.

Continuous variables were compared by using a Mann-Whitney test.

$P < 0.05$ was considered statistically significant for all analyses.

High neutralizing titer group was defined as participants with top 30% of neutralizing titers at day 14 post booster vaccination.

Low neutralizing titer group was defined as participants with bottom 30% of neutralizing titers at day 14 post booster vaccination.

BMI= Body Mass Index, OR= Odds ratio

Supplementary information, Table S3. Solicited and unsolicited adverse reactions

	**Day0-Day3**	**Day4-Day14**	**Day15-Day28**
**Solicited Injection Site Adverse Reactions**			
Any (%)	30 (42.3%)	4 (5.6%)	1 (1.4%)
Grade 1	26 (36.6%)	3 (4.2%)	1 (1.4%)
Grade 2	4 (5.6%)	1 (1.4%)	0 (0.0%)
Pain	20 (28.2%)	1 (1.4%)	1 (1.4%)
Grade 1	18 (25.4%)	0 (0.0%)	1 (1.4%)
Grade 2	2 (2.8%)	1 (1.4%)	0 (0.0%)
Erythema	3 (4.2%)	1 (1.4%)	0 (0.0%)
Grade 1	3 (4.2%)	1 (1.4%)	0 (0.0%)
Induration	3 (4.2%)	0 (0.0%)	0 (0.0%)
Grade 1	3 (4.2%)	0 (0.0%)	0 (0.0%)
Pruritus	4 (5.6%)	1 (1.4%)	0 (0.0%)
Grade 1	4 (5.6%)	1 (1.4%)	0 (0.0%)
Swelling	4 (5.6%)	1 (1.4%)	0 (0.0%)
Grade 1	4 (5.6%)	1 (1.4%)	0 (0.0%)
**Solicited Systematic Adverse Reactions**			
Any (%)	8 (11.3%)	2 (2.8%)	1 (1.4%)
Grade 1	6 (8.5%)	2 (2.8%)	1 (1.4%)
Grade 2	2 (2.8%)	0 (0.0%)	0 (0.0%)
Fever	1 (1.4%)	0 (0.0%)	0 (0.0%)
Grade 1	1 (1.4%)	0 (0.0%)	0 (0.0%)
Fatigue	6 (8.5%)	1 (1.4%)	1 (1.4%)
Grade 1	5 (7.0%)	1 (1.4%)	1 (1.4%)
Grade 2	1 (1.4%)	0 (0.0%)	0 (0.0%)
Myalgia	1 (1.4%)	0 (0.0%)	0 (0.0%)

Grade 2	1 (1.4%)	0 (0.0%)	0 (0.0%)
Rash	1 (1.4%)	1 (1.4%)	0 (0.0%)
Grade 1	1 (1.4%)	1 (1.4%)	0 (0.0%)
Cough	0 (0.0%)	0 (0.0%)	1 (1.4%)
Grade 1	0 (0.0%)	0 (0.0%)	1 (1.4%)
Anorexia	0 (0.0%)	0 (0.0%)	1 (1.4%)
Grade 1	0 (0.0%)	0 (0.0%)	1 (1.4%)
Arthralgia	0 (0.0%)	0 (0.0%)	0 (0.0%)
Dyspnea	0 (0.0%)	0 (0.0%)	0 (0.0%)
Nausea	0 (0.0%)	0 (0.0%)	0 (0.0%)
Pharyngalgia	0 (0.0%)	0 (0.0%)	0 (0.0%)
Syncope	0 (0.0%)	0 (0.0%)	0 (0.0%)
Vertigo	0 (0.0%)	0 (0.0%)	0 (0.0%)
Vomiting	0 (0.0%)	0 (0.0%)	0 (0.0%)
**Unsolicited Injection Site Adverse Reactions**			
Any (%)	0 (0.0%)	0 (0.0%)	0 (0.0%)
Grade 1	0 (0.0%)	0 (0.0%)	0 (0.0%)
**Unsolicited Systematic Adverse Reactions**			
Any (%)	5 (7.0%)	0 (0.0%)	0 (0.0%)
Grade 1	5 (7.0%)	0 (0.0%)	0 (0.0%)
Stomachache	1 (1.4%)	0 (0.0%)	0 (0.0%)
Grade 1	1 (1.4%)	0 (0.0%)	0 (0.0%)
Headache	1 (1.4%)	0 (0.0%)	0 (0.0%)
Grade 1	1 (1.4%)	0 (0.0%)	0 (0.0%)
Sneezing	1 (1.4%)	0 (0.0%)	0 (0.0%)
Grade 1	1 (1.4%)	0 (0.0%)	0 (0.0%)
Lethargy	1 (1.4%)	0 (0.0%)	0 (0.0%)
Grade 1	1 (1.4%)	0 (0.0%)	0 (0.0%)

Hand Trembling	1 (1.4%)	0 (0.0%)	0 (0.0%)
Grade 1	1 (1.4%)	0 (0.0%)	0 (0.0%)

Study protocol

The study was conducted complying the following study protocol. Written informed consent was obtained before the enrollment. The study protocol and informed consent form were approved by the Ethics Committee of Huashan Hospital.

Inclusion and Exclusion Criteria

Inclusion criteria:

1. Healthy adults, or adults with pre-existing but stable medical conditions (Participants didn’t require significant change in therapy or hospitalization within 3 months before enrollment).
2. Participants who have received priming vaccination by two doses of inactivated whole-virion vaccines (CoronaVac or BBIBP-CorV) within 4 to 8 months.
3. Participants that are willingly to comply with the study procedures and provide written informed consent.

Exclusion criteria:

1. SARS-CoV-2 infection confirmed by positive reverse transcription-polymerase-chain-reaction (RT-PCR) assay.

2. A history of infection with SARS-CoV-2, or a history of contacting with cases of confirmed or suspected SARS-CoV-2 infection.

3. Presence of fever, cough, runny nose, sore throat, diarrhoea, dyspnoea, or tachypnoea within 7 days before screening visit.

4. Allergy to any ingredient included in SARS-CoV-2 vaccines.

5. A history of severe allergy (such as angioneurotic oedema or allergic shock) to any vaccination.

6. A positive blood pregnancy test.

7. A history or family history of mental illness or serious central nervous system diseases (such as epilepsy, transverse myelitis, Guillain Barre syndrome, demyelinating disease, encephalopathy etc.).

8. Suffering from severe liver or kidney disease.

9. Uncontrollable hypertension (systolic blood pressure over 180 mmHg, diastolic blood pressure over 100 mmHg).

10. Diabetes complications.

11. Malignant tumors.

12. Other acute diseases attack or chronic diseases with acute exacerbation.

13. Known history of cancer or solid organ transplant.

14. Known immunosuppressive or immunodeficient state including confirmed HIV infection, and history of receiving systemic immunosuppressants within 3 months prior to the day of screening.

Outcomes

The primary endpoint was the increased geometric mean titer (GMT) of pseudovirus neutralizing antibodies against SARS-CoV-2 and VOCs on day 14 after the boosting dose. The secondary outcomes included increased anti-RBD responses and T cell-mediated responses on day 14 and day 28 after the boosting dose, as well as the occurrence of adverse reactions within 28 days after the boosting dose.

Assessment of Safety

Solicited systemic and local adverse reactions were recorded by the participants on designed electronic questionnaires. Questionnaires were distributed and collected by trained site personnel at day 3, day 14 and day 28 after the boosting dose.

Plasma surrogate virus neutralization test (sVNT), anti-RBD antibody and IgG test

Blood samples were taken from participants for serology tests by day 0, 14 and 28 after the boosting dose. We assessed the anti-RBD responses induced by a third boosting vaccination. Plasma sVNT titer was determined by using a SARS-CoV-2 Neutralizing Ab detection kit (PerkinElmer SuperFlex Anti-SARS-CoV-2 Neutralizing Ab Kit, SDX-57042) following manufacturer's protocol. The anti-RBD antibody and IgG was measured by PerkinElmer SuperFlex Anti-SARS-CoV-2 Ab Kit and SuperFlex Anti-SARS-CoV-2 IgG Kit. According to the manufactory brochures (www.perkinelmer.com), we used superparamagnetic microparticles together with direct chemiluminescence technology to detect antibody in plasma samples. Plasma was serial diluted before detection, 50μl diluted sample was added to sample wells, and then mixed with 50ul SARS-CoV-2 receptor binding domain (RBD) protein labeled with acridinium ester. Signals were captured using PerkinElmer SuperFlex automatic chemiluminescence immunoassay analyzer.

To measure the neutralizing titer, the signals were converted to sVNT titer using a reference standard curve plotted with kit-suppled reagents. The sVNT titer was determined by the reciprocal of the last dilution that resulted in >50% reduction of chemiluminescence signal.

To measure the anti-RBD antibody, s sample was added to a sample well, and then bounded with the magnetic particles coated with SARS-CoV-2 antigen. Together with biotinylated anti-RBD SARS-CoV-2 antigen, an immunocomplex was formed. After washing, an acridinium ester-labeled anti-biotin antibody was added to form a new immunocomplex. Unbound substances were removed by washing and the luminescence value of the chemiluminescence reaction was measured under the action of pre-trigger and trigger solution. The luminous intensity was positively correlated with the concentration of the anti-SARS-CoV-2 antibody in the sample.

To measure the anti-RBD IgG, a specimen was added to a specimen well, and then bounded with the magnetic particles coated with SARS-CoV-2 antigen. After washing, acridinium ester labeled anti-human IgG antibody was added to form an immunocomplex. Unbound substances were removed by washing and the luminescence value of the chemiluminescence reaction was measured under the action of pre-trigger and trigger solution. The luminous intensity was positively correlated with the concentration of the Anti-SARS-CoV-2 IgG in the specimen.

The antibody assay was analyzed in its original scale, and results were then converted to the WHO international standard units using the conversion factors supplied by the laboratory.

Plasma pseudovirus neutralization test (pVNT)

Blood samples were taken from participants for serology tests at days 0 and 14. Pseudovirus incorporating with spike protein from either SARS-CoV-2, variants (Alpha, Beta, Gamma and Delta) were constructed using a procedure described by Nie et al[1]. On day before transfection, 293T cells were prepared and adjusted to the concentration of 5-7 x 10^5cell/mL with DMEM complete medium. 30μg of plasmid pcDNA3.1.VSVG (or plamid pcDNA3.1.S2), which expressing the spike protein was transfected according to the instruction. Afterwards, 15mL of diluted G*ΔG-VSV (VSV G pseudovirus) at concentration of 7.0 x 10^4 TCID50/mL was added into T75 flask, thus the transfected cells were subsequently infected. The 50% tissue culture infectious dose (TCID50) of SARS-CoV-2 pseudovirus was determined using a single-use aliquot from the pseudovirus bank to avoid inconsistencies resulted from repeated freezing-thawing cycles.

The 96-well flat-bottom culture plates were used to titration the pseudovirus. A 2-fold initial dilution with six replicates was made, followed by serial 3-fold dilutions, and the last column was used as the cells control without pseudovirus. Subsequently, cells were adjusted to $2x10^5$ cells/mL with DMEM complete medium, and 100μl of cell suspension was added to each well. After 2-min incubation in darkness at room temperature, the cell completed lysis, and 150μl of lysate was transferred to 96-well chemiluminescence detection plates (PerkinElmer, Ensight) for detection. Positive was determined to be ten-fold higher than the negative (cells only) in terms of relative luminescence unit (RLU) values. TCID50 was calculated using the Reed–Muench method.

The virus neutralization assay was conducted as follows: 100 μl serial dilutions of human sera or monoclonal antibody preparations were added into 96-well plates[1]. After that, 50 μl pseudoviruses with concentration of 1300 TCID50/mL were added into the plates, followed by incubation at 37°C for 1 hour. Afterwards, HuH-7 cells were added into the plates (2×104 cells/100 μl cells per well), followed by incubation at 37°C in a humidified atmosphere with 5% CO2. Chemiluminescence detection was performed after 24 hours incubation. The Reed-Muench method was used to calculate the virus neutralization titer. The results are based on 3-5 replicates unless specified. In order to validate the test operation process, the Coefficient of Variance (CV) control of replicates is set within 30% of six wells, so is the CV for the duplicate sample wells.

Detection of S and N protein specific cellular immune response

ELISpot assays were performed to evaluate SARS-CoV-2 specific T-cell response using Human IFN-gamma ELISpot kit (Fosun Pharma, Shanghai, China). The peripheral blood mononuclear cells (PBMC) were stimulated by S1, S2 and N peptide pools for 20 hours at 37℃ with 5% CO_2. Phytohemagglutinin was added as a positive control, and cells cultured without stimulations were used as a negative control. After incubation, detection antibody was added according to the manufactures' s instruction. We counted IFN-γ producing spots by ELISpot Reader (version 7.0). Then the IFN-γ spot-forming units (SFU) per million PBMC was calculated.

Statistical analysis

We present summary statistics for individuals as median with inter-quartile ranges (IQRs), or geometric mean with 95% confidence intervals (CIs). Adverse reactions post vaccination

were expressed as numbers and proportions. Mann-Whitney U test, Student's t test or One-way ANOVA test was used for continuous variables, and Pearson χ^2 test or Fisher's exact test for categorical variables to assess the statistical significance between groups and subgroups. Hypothesis testing was two-sided and P values of less than 0.05 was considered to be significant. IBM SPSS (version 20.0) and Graphpad Prism (version 9.2) were used for statistical analysis.

Reference:

1. Nie J, Li Q, Wu J, et al. Establishment and validation of a pseudovirus neutralization assay for SARS-CoV-2. Emerg Microbes Infect. 2020;9(1):680-686.

Supplementary Dataset

Subgroup analysis of participants received priming vaccination by two doses of CoronaVac or BBIBP-CorV

1) **Sub-group analysis of booster group: priming vaccination by two doses of inactivated vaccine (Whole Booster Group or BBIBBP-CorV Group)**

Subgroup Analysis of Figure 1a and 1f: T cell responses of Booster Group on Day 0, 14 days after Day 0 and 28 days after Day 0. (Median [IQR])

		Whole Booster group (n=71)	BBIBP-CorV Booster Group (n=69)	*P* value
**Day0**	S1	10 (0-20)	10(0-20)	0.9708
	S2	5 (0-20)	5 (0-20)	0.9725
	N	10 (0-25)	10 (0-25)	0.9508
**Day14**	S1	5(0-15)	5(0-15)	0.8297
	S2	10(5-50)	10(5-45)	0.9046
	N	5(0-20)	5(0-20)	0.8502
**Day28**	S1	5(0-5)	5(0-5)	0.8681
	S2	10(0-25)	5(0-25)	0.9003
	N	10(5-25)	10(5-25)	0.8993

Subgroup Analysis of Figure 1b and 1l: pVNT : Day 0 (GMT [95% CI])

	Whole Booster group (n=71)	BBIBP-CorV Booster Group (n=69)	*P* value
**Wuhan-Hu-1**	24.89(20.63,30.02)	25.07(20.64,30.46)	0.9564
**Alpha**	15.78(13.1,19.00)	15.77(13.01,19.11)	0.9967
**Beta**	10.65(9.19,12.35)	10.59(9.07,12.35)	0.9562
**Gamma**	16.79(14.15,19.91)	17.24(14.49,20.52)	0.8274
**Delta**	22.56(18.89,26.96)	22.47(18.68,27.03)	0.9747

Subgroup Analysis of Figure 1g and 1l: pVNT :14 days after Day 0 (GMT [95% CI])

	Whole Booster group (n=71)	BBIBP-CorV Booster Group (n=69)	*P* value
**Wuhan-Hu-1**	1881.01(1375.56,2572.19)	1865.80(1346.87,2584.68)	0.9517
**Alpha**	1523.21(1100.68,2107.95)	1513.38(1079.37,2121.9)	0.9781
**Beta**	785.92(543.66,1136.12)	773.55(527.85,1133.61)	0.9526
**Gamma**	1307.73(921.14,1856.56)	1288.62(895.63,1854.06)	0.9537
**Delta**	1944.15(1403.33,2693.39)	1882.68(1342.72,2639.76)	0.8916

Subgroup Analysis of Figure 1c and 1h: sVNT :(GMT [95% CI])

sVNT	**Whole Booster group (n=71)**	**BBIBP-CorV Booster Group (n=69)**	***P* value**
**Day 0**	18.19(13.12,25.20)	18.29(13.03,25.68)	0.9812
**14 days after Day 0**	9157.83(6588.84,12728.49)	8845.96(6289.65,12441.23)	0.8843
**28 days after Day 0**	8159.68(5927.73,11232.03)	8216.87(5885.71,11471.32)	0.9760

Subgroup Analysis of Figure 1d and 1i: Anti-RBD antibody (IQR)

	**Whole Booster group (n=71)**	**BBIBP-CorV Booster Group (n=69)**	***P* value**
**Day 0**	8.47(2.84,30.06)	8.32(2.95,25.41)	0.9141
**14 days after Day 0**	8342.00(3223.87,22350.00)	8334.00(3244.75,22290)	0.9795
**28 days after Day 0**	4020.50(950.30,13050.00)	4047.00(888.95,13100)	0.9762

Subgroup Analysis of Figure 1e and 1j: Anti-RBD IgG (IQR)

	**Whole Booster group (n=71)**	**BBIBP-CorV Booster Group (n=69)**	***P* value**
**Day 0**	4.42(2.09,12.41)	4.38(1.97,12.85)	0.9369
**14 days after Day 0**	1218.50(869.75,1444.00)	1212.00(856.35,1453.50)	0.9729
**28 days after Day 0**	1140.50(786.60,1446.00)	1110.00(771.70,1448.50)	0.9238

2) Sub-group analysis of control group: priming vaccination by two doses of inactivated vaccine (Whole Control Group or BBIBBP-CorV or CoronaVac)

Subgroup Analysis of Figure 1a: Baseline IFN-γ SFU/ million PBMCs against S1, S2 and N peptide at 4-8 months after the second dose. (Median [IQR])

	**Booster Group (n=71)**	**Whole Control Group (n=51)**	***P* value**
**S1**	10 (0-20)	5 (0-10)	0.1134
**S2**	5 (0-20)	0 (0-10)	0.0442
**N**	10 (0-25)	15 (5-35)	0.0584

	**Booster Group (n=71)**	**CoronaVac Control Group (n=11)**	***P* value**
**S1**	10 (0-20)	5 (0-25)	0.9888
**S2**	5 (0-20)	5(0-25)	0.9719
**N**	10 (0-25)	20(0-25)	0.6975

	**Booster Group (n=71)**	**BBIBBP-CorV Control Group (n=40)**	***P* value**
**S1**	10 (0-20)	2.5(0-10)	0.0603
**S2**	5 (0-20)	0(0-5)	0.0180
**N**	10 (0-25)	15(5-35)	0.0390

Subgroup Analysis of Figure 1b: Baseline antibody response 4-8 months after second dose evaluated by pVNT (GMT [95% CI])

	**Booster Group (n=71)**	**Whole Control Group (n=51)**	**BBIBBP-CorV Control Group (n=40)**	**CoronaVac Control Group (n=11)**
**Day 0**	24.89(20.60,30.02)	26.44(21.20,33.00)	25(19.00,32.89)	32.48(24.72,42.67)

***P* value**	**Whole Control Group (n=51)**	**Control Group-BBIBBP-CorV Control Group (n=40)**	**Control Group-CoronaVac Control Group (n=11)**
**Booster Group (n=71)**	0.676	0.979	0.098

Subgroup Analysis of Figure 1c: Baseline antibody response 4-8 months after second dose evaluated by sVNT (GMT [95% CI])

sVNT	**Booster Group (n=71)**	**Whole Control Group (n=51)**	**Control Group-BBIBBP-CorV Control Group (n=40)**	**Control Group-CoronaVac Control Group (n=11)**
**Day 0**	18.19(13.12,25.20)	31.57(22.55,44.20)	25.55(17.41,37.49)	68.16(38.6,120.36)

***P* value**	**Whole Control Group (n=51)**	**Control Group-BBIBBP-CorV Control Group (n=40)**	**Control Group-CoronaVac Control Group (n=11)**
**Booster Group (n=71)**	0.189	0.422	0.096

Subgroup Analysis of Figure 1d: Baseline antibody response 4-8 months after second dose evaluated by total Anti-RBD antibody (IQR)

	Booster Group (n=71)	Whole Control Group (n=51)	BBIBBP-CorV Control Group (n=40)	CoronaVac Control Group (n=11)
**Day 0**	8.47(2.84,30.06)	22.14(9.13,33.60)	19.56(6.58,32.87)	25.79(17.86,48.53)

***P* value**	**Whole Control Group (n=51)**	**BBIBBP-CorV Control Group (n=40)**	**CoronaVac Control Group (n=11)**
**Booster Group (n=71)**	0.033	0.171	0.010

Subgroup Analysis of Figure 1e: Baseline antibody response 4-8 months after second dose evaluated by Anti-RBD IgG (IQR)

	Booster Group (n=71)	Whole Control Group (n=51)	BBIBBP-CorV Control Group (n=40)	CoronaVac Control Group (n=11)
**Day 0**	4.42(2.09,12.41)	11.19(4.28,21.06)	9.98(3.09,18.10)	17.61(10.60,57.55)

***P* value**	**Whole Control Group (n=51)**	**BBIBBP-CorV Control Group (n=40)**	**CoronaVac Control Group (n=11)**
**Booster Group (n=71)**	0.031	0.061	0.002

3) Sub-group analysis of Control groups at Day 0，14 days after Day 0 and 28 days after Day 0: priming vaccination by two doses of inactivated vaccine (Whole Control Group or BBIBBP-CorV or CoronaVac)

Subgroup Analysis of Figure 1h: Antibody response in boost group evaluated by sVNT (GMT [95% CI])

	**Day 0**	**14 days after Day 0**	**28 days after Day 0**	***P* value**
**Whole Control Group (n=51)**	31.57(22.55,44.20)	29.90(20.73,43.12)	29.49(20.92,41.59)	0.9159
**BBIBBP-CorV Control Group (n=40)**	25.55(17.41,37.49)	23.43(15.26,35.97)	25.55(17.95,36.35)	0.9982
**CoronaVac Control Group (n=11)**	68.16(38.6,120.36)	72.6(48.33,109.05)	49.74(17.83,138.77)	0.8520

Subgroup Analysis of Figure 1i: Antibody response in boost group evaluated by total Anti-RBD antibody (IQR)

	**Day 0**	**14 days after Day 0**	**28 days after Day 0**	***P* value**
**Whole Control Group (n=51)**	22.14(9.13,33.60)	14.78(5.84,22.26)	19.11(8.56,33.33)	0.1774
**BBIBBP-CorV Control Group (n=40)**	19.56(6.58,32.87)	13.04(4.22,21.28)	17.41(4.09,31.54)	0.2383
**CoronaVac Control Group (n=11)**	25.79(17.86,48.53)	19.74(18.16,28.78)	31.85(17.08,42.59)	0.5624

Subgroup Analysis of Figure 1j : Antibody response in boost group evaluated by Anti-RBD IgG (IQR)

	**Day 0**	**14 days after Day 0**	**28 days after Day 0**	***P* value**
**Whole Control Group (n=51)**	11.19(4.28,21.06)	7.41(3.18,12.34)	11.47(4.1,17.01)	0.0803
**BBIBBP-CorV Control Group (n=40)**	9.98(3.09,18.10)	6.77(2.46,10.18)	10.34(3.91,14.77)	0.0503
**CoronaVac Control Group (n=11)**	17.61(10.60,57.55)	12.96(6.99,41.01)	15.13(7.59,31.07)	0.4932
